# Case of a Fracture of the Sternum

**Published:** 1787

**Authors:** John Hale

**Affiliations:** Surgeon to the New Finsbury Dispensary


V. *Caſe of a Fracture of the Sternum. By Mr.* John Hale, *Surgeon to the New Finſbury Diſpenſary.*

ALTHOUGH fractures of the ſternum are deſcribed by every writer on the accidents to which the bones are ſubject, yet they appear to have ſeldom occurred, even to the oldeſt practitioner. All thoſe authors whom I have had an opportunity of conſulting, very fully deſcribe in what manner this accident may happen, and as fully preſcribe the rules to be obſerved obferved in the treatment of it; but they feem to have copied from each other, without adducing any proofs to corroborate their affertions. Du Verney, in his excellent Treatife on the Difeafes of the Bones, is the only writer (that I have met with) who enters fully and circumftantially into fractures of this part. He mentions three cafes which had occurred to him, two of which proved inftantly fatal, and the third recovered, but the patient remained an invalid during the reft of his life. He likewife afferts, that "all thefe difeafes are mor-"tal; to fome at the inftant of the blow or "fall, and to others fome days after: never-"thelefs," he adds, "there have been pa-"tients whofe conftitutions have held out, and "have been cured *." I can only regret that he gives no information where thofe cafes are recorded.

### C A S E.

John Oates, aged thirty-eight, by trade a cabinet-maker, being in company with fome

* Englifh Tranflation of M. Du Verney's Treatife. 8vo. London, 1762: p. 92.

friends friends at a tavern, and drinking freely, a diſpute aroſe, which terminated in blows, and he was unfortunately thrown down with ſuch violence on the edge of a chair, as to cauſe a fracture of the ſternum, about four inches above the enſiform cartilage.

Being carried home, a neighbouring apothecary was ſent for, who ordered ſome draughts to be taken, and a volatile liniment to embrocate the part. The ſymptoms, however, ſoon became alarming; theſe were great pain and difficulty in reſpiration, cough, and oppreſſion on the lungs. Theſe complaints were ſuffered to increaſe, without any means being uſed to alleviate them, for the ſpace of a week; although he often ſpoke with certainty of his breaſt being broken, and ſaid he could "feel "the bones jar againſt each other."

Obtaining no relief, and being poſitively told that his complaint was only a bruiſe, he diſmiſſed his apothecary, and applied to the New Finſbury Diſpenſary, on Tueſday, the 23d of January, 1787. He was immediately viſited by Dr. Skeete and myſelf, with Mr. Haighton, who happened to call on me that morning. On examination, we perceived that a fracture had taken place as deſcribed above, but how much

 of of the bone was depreſſed, or how deep the depreſſion might be, we could not readily aſcertain, owing to an inflammation which occupied the inferior and middle part of the ſternum, and which ſeemed haſtily advancing to ſuppuration. His pulſe was hard and full, the cough frequent, and attended with ſuch diſtreſs and anxiety to the patient, that he ſaid he ſhould be perfectly well if the cough could be ſtopped; for at that time he could plainly feel the broken edges of the bone grate on each other, the irritation of which was almoſt inſupportable, and prevented his taking any reſt.

We immediately took away ſixteen ounces of blood, which was much inflamed. A laxative mixture was ordered to be taken occaſionally; an emulſion of ſpermaceti, with nitre and tinct. thebaic, every three hours; and every other means was uſed to obviate the effects which we imagined would be the conſequences of ſuch an accident. On the day following the pulſe was rather ſofter, and the reſpiration not quite ſo laborious. Ten ounces more of blood were taken from him, which had the ſame inflammatory appearance as before. The inflammation on the ſternum was now more circumſcribed, and evidently ſhewed that ſome pus was was already formed. The laxative medicine was ordered to be repeated, and thirty drops of tinct. thebaic. to be taken at night.

The ſymptoms continued much the ſame till Saturday, the 27th of January, when Mr. Cooper, Surgeon to Guy's Hoſpital, favoured me with his company, and was of opinion that the abſceſs on the ſternum ſhould be immediately opened. About ſix ounces of pus were diſcharged. On paſſing my finger to the bottom of the wound, I expected to have diſcovered the broken portion of bone, but did not, probably owing to the parts having become thickened in conſequence of the preceding inflammation. The fracture, however, was evident to one ſenſe, though not to the other; for, on the patient deſiring us to place our ear on the wound, we could hear the edges of the bone grate on each other at every inſpiration. The pulſe being ſtill full, it was thought neceſſary to take away ſix ounces more of blood. He had no reſt this night, the cough being inceſſant.

On Sunday, the 28th, his countenance appeared much dejected, the ſkin hot, but the pulſe calm; I ordered the opiate to be increaſed to ſeventy drops of tinct. thebaic. and applied a bandage, or rather a girt, (ſuch as I uſe in fractures of the ribs, and which is made to buckle to the degree of tightnefs that may be requifite) in hopes of being able to confine the motion of the fternum, and thereby prevent the irritation which the fracture produced. I intended to have applied this bandage when I firft vifited him, but it was thought that if much preffure was thrown on the ribs, it might probably caufe the inferior broken part of the bone to protrude through the inflamed integuments, and be productive of much danger; the bandage was therefore deferred till the abfcefs was opened, and the bone not being evident to the touch, we had lefs to fear from the application of a moderate preffure. He experienced much relief in confequence of the bandage being applied, the cough was greatly abated, the opiate procured a found fleep, and the bone was not troublefome in infpiration; the pulfe was perfectly calm, and the wound difcharged a healthy pus. He was now ordered fome mutton broth, having hitherto been confined to water-gruel.

From this time, to Monday the 6th of February, the fymptoms indicated much danger; he fweated profufely, coughed inceffantly, and what he expectorated was often tinged with blood. He took liberally of a decoction of bark with elixir of vitriol, drank warm milk every morning, and ufed fuch nourifhing articles of diet as were directed by Dr. Skeete. This plan feemed to fucceed, for, from the 6th to the 12th of February, the fweats were much diminifhed, and the cough lefs frequent; but the wound difcharged a fœtid ichor in great abundance, which proceeded from a finus extending above and for fome fpace round the fuperior part of the wound. We were now of opinion that this ichorous difcharge might be occafioned by the edges of the broken bone; if fo, it became advifable to make a farther opening, to endeavour to reach the foundation of the mifchief, and then purfue fuch a courfe as might be indicated from examining the condition of the parts.

This, however, was delayed till Saturday the 17th of February, as for fome days we had pleafed ourfelves with an idea that the parts would unite independently of our interference, efpecially as the difcharge from the wound was become thicker, (Dr. Skeete having added gum myrrh to the decoction of bark); but we were unhappily difappointed in our expectations; and as no time was to be loft, I informed the patient of the plan which, on consultation, we had agreed to follow, viz. to make an incision down to the bone, to examine its condition, and, if necessary, to remove whatever part of it might, in our opinion, be an obstacle to a perfect union. He readily assented to whatever might be judged expedient for his relief; not on his own account, having suffered so much that life was become a burden, but for the sake of his wife and five infant children, whose subsistence depended solely on his labour.

I made an incision from about an inch above the edge of the old wound, and continued it to the bottom; the knife grated on the bone, and the inferior part became evident to the touch, though covered externally with a smooth granulation. It now appeared that the direction of the fracture was not strictly transverse, but a little oblique, being somewhat lower on the right side than on the left; but the natural junction of the cartilages of the ribs, immediately above and below the fractured part, remained entire. I passed my finger into the cavity made by the dilatation, and felt the internal surface of the sternum rough and irregular. We now could only conjecture that some degree of exfoliation would ensue; but in all probability the consequence of this operation operation might prove fatal by caufing that irritation often confequent to the expofure of cavities. Theory certainly juftified us in thefe conclufions; but practice has happily convinced us that, notwithftanding thefe difcouragements, there was ftill a method left to prevent what we had fo much reafon to fear. The fractured portions of the bone were not denuded in every part; both the fuperior and inferior portions were covered with granulations: it then became an object to endeavour to promote an union of thofe parts by incarnation, fimilar to what occurs in compound fractures, in which light it might now certainly be confidered.

I muft here obferve, that on the day following I found him much better, he became eafier after the operation, coughed but little, and flept the greateft part of the night without taking the opiate. This was the firft natural fleep he had enjoyed fince the accident happened, for the opiates had only ferved to palliate the cough, without procuring any refrefhing fleep, although he generally took one hundred drops of tinct. thebaic. The expectoration alfo appeared more frothy, and he had no difficulty in difcharging it from the lungs. This amendment however, was but temporary; for, from the 17th to to the 24th of February, the opiates were again obliged to be given. Our attempts to procure an union of the parts proved abortive, and the inferior edge of the bone protruded through the wound at every inſpiration, and conſequently expoſed that cavity which the preceding inflammation and preſſure had undoubtedly produced.

There ſeemed no proſpect of any exfoliation; I therefore omitted the former bandage, as it appeared to have no power to anſwer the intention we had in view, and paſſed a common roller round the inferior part of the ſternum; the preſſure of this brought the lower portion in contact with the upper, and we had the pleaſure of ſeeing an union of the ſoft parts effected in a few days. The ſweats from this time totally ceaſed, and the cough was not troubleſome. I removed him to Iſlington for the benefit of the air; there the cough entirely left him, and he ſlept without taking the opiate.

When he attempted a deep inſpiration, he felt a ſmall preſſure on the fractured part, and alſo on the right ſide of the wound; which ſide was apparently enlarged, and felt as if the cartilages of the ribs had received ſome injury from the fall; but upon a more attentive examination mination was found to be owing to a depreffion of the upper part of the fternum, together with the cartilages of the ribs. He complained that the air was too fharp for him, and (to ufe his own expreffion) "felt like vinegar in his "noftrils;" indeed this was fo much the cafe, that if he ftood at the window when open, or attempted to walk out, the air inftantly caufed him to fneeze violently, which gave great pain to the fractured part. I was therefore obliged to remove him from Iflington, where he only continued four days; and for two or three nights after he returned home the cough again became violent, fo that I was under the neceffity of repeating the opiates. As the wound healed the cough abated, and his only complaint was that of great weaknefs, and a total inability to ufe his arms with any freedom of motion. He could ftoop to buckle his fhoe, and could take up any weight, but was utterly unable to perform any lateral motion.

During the month of March he was perfectly free from complaint. The enlargement, related to have been on the right fide of the wound, was much leffened, but an abfcefs formed on that fide, immediately on the breaft, which broke on Friday the 30th of March. It ap-

 peared peared quite superficial, and to have had no connexion with the original injury.

In April, the wound on the sternum was healed to about the size of a sixpence, and from this a vast discharge was daily evacuated. Suspecting some sinus, I passed the probe, and found one, which extended about two inches on the right side between the two inferior cartilages, directly above the enlargement already mentioned. This sinus was laid open, and continued discharging till the beginning of August, when it was proposed to apply a caustic on the part, from the action of which I not only expected the exfoliation of any diseased part to be facilitated, but also a greater degree of excitement in the adjacent living parts for the purpose of generating a bony union. The event fully answered my expectation, and the action of the caustic laid bare the fractured portions of the bone, the superior edge of which was greatly depressed, and the intermediate space filled with a kind of ligamentous substance, but without any ossific union of the parts. It was curious to observe the process which nature was daily executing, in order to fill up the vacuity; at every dressing some new excitement was perceived; increased vascularity rity * in the living portions of bone, by which the carious part became more feparated; and granulations arifing from the furrounding foft parts. On the 10th of October the exfoliation from the inferior part took place; the portion of bone was but fmall, healthy granulations being fubftituted in its place.

We affifted the patient with an elaftic trufs, which proved of fo much fervice as to enable him again to follow his bufinefs of a cabinet-maker. This trufs makes an equal preffure on each fide of the breaft, and fupports the fternum in fuch a manner, that if he takes it off, he fays, he feels "as if his infide is falling in "pieces." Without it, he is incapable of performing any lateral motion.

* In order more fully to explain what I mean by an "increafed vafcularity," it is neceffary to obferve, that when the flough (which the action of the cauftic had produced) was feparated from the fternum, a very large portion of the inferior part of the bone appeared to be entirely deprived of the living principle; but fortunately this was not the cafe: every day's infpection fully proved it, by that portion becoming more and more florid; and by the affiftance of a glafs we could plainly difcover new veffels fhooting in a tranfverfe direction through the offeous fibres. I therefore truft that the term of "increafed vafcularity" is not mifapplied, and I am at a lofs for any other to exprefs my meaning more intelligibly.

 The

The wound healed, without any interruption, and was perfectly cicatrized on the 23d of October.

The following obfervations are fuch as will naturally fuggeft themfelves on an attentive confideration of the above facts:

Firft, That the cafe was attended with confiderable danger throughout the greater part of its progrefs, but from very different and even oppofite circumftances at different periods. In the beginning, the fymptoms of inflammation in the thorax feemed to be fo general and violent, that without the free ufe of the lancet, affifted by dilution and the antiphlogiftic regimen, it is probable that a fatal termination would fpeedily have enfued; but the only method by which this could be prevented, doubtlefs, affifted in inducing that ftate of debility and hectic fever, under which the patient was afterwards in danger of finking, and which required the liberal exhibition of bark, elixir of vitriol, myrrh, and fuch nourifhing articles of diet as were judicioufly prefcribed by Dr. Skeete. The fame thing happens in many cafes of difeafe which fall under the care of the furgeon; and the prefent inftance is a ftrong illuftration of the neceffity of a particular attention being required to the ſtate of the conſtitution, as well as to the condition of the diſeaſed part.

Secondly, It may appear extraordinary, that notwithſtanding the frequent expoſure of a cavity, which appeared when the broken edges of the bone were ſeparated, during the alternate motions of inſpiration and expiration, that this expoſure was not followed by any repeated attack of inflammation, or by any effuſion of pus into the cavities of the thorax; though we had more than once ſufficient reaſon to apprehend ſuch effects, eſpecially when the expectoration aſſumed a purulent and bloody appearance, with ſymptoms of hectic fever. The event, therefore, ſeems to prove that adheſions muſt have taken place between the lungs and pleura, in the neighbourhood of the injured part, ſo as to have completely prevented any ſuch effect.

Thirdly, After ſome months the treatment was almoſt entirely confined to the part; the healing of the wound having been prevented by the ſtate of the bone underneath, as has been already mentioned.

Fourthly, From the ſenſation which the patient experiences at preſent, I have every reaſon to conclude that the loſs of ſubſtance, which the ſternum has ſuſtained, is ſupplied by granulation lation only; and whether nature will in this caſe, as in many others, reſtore the part to its original ſtate, by a bony depoſition, time alone will be able to diſcover.

*October*, 1787.

P. S. Since the foregoing account went to the preſs, I have read a caſe of fractured ſternum, in the Edinburgh Medical Commentaries *, by Mr. George Borthwick, Surgeon to the fourteenth regiment of Dragoons. In many inſtances the ſymptoms agreed with thoſe mentioned in the caſe I have deſcribed, particularly the grating noiſe in the time of reſpiration. Mr. Borthwick having been immediately called in, prevented the train of dangerous ſymptoms which unfortunately befel my patient. The account being drawn up with great preciſion, I refer my readers to the book itſelf for farther information.

The addition of this poſtſcript affords me an opportunity of ſaying, that my patient called on me at the Diſpenſary, on the 30th ult. and informed me he had gained ſo much ſtrength within a few days, as to be enabled to

* Vol. IV. p. 185.

perform perform the moſt laborious part of his work without the aſſiſtance of the truſs.

*November*, 1787.

